# Hsa_circ_0001879 promotes the progression of atherosclerosis by regulating the proliferation and migration of oxidation of low density lipoprotein (ox-LDL)-induced vascular endothelial cells via the miR-6873-5p-HDAC9 axis

**DOI:** 10.1080/21655979.2021.1997224

**Published:** 2021-12-07

**Authors:** Feifei Li, Yahui Chen, Zhiling He, Chuangchang Wang, Xiaoli Wang, Guangming Pan, Jiang yang Peng, Qiuxiong Chen, Xia Wang

**Affiliations:** a Department of Cardiology, Guangdong Provincial Hospital of Traditional Chinese Medicine; The Second Affiliated Hospital of Guangzhou University of Chinese Medicine, Guangzhou, Guangdong, China; b The Second Clinical College of Guangzhou University of Chinese Medicine, Guangzhou, Guangdong, China

**Keywords:** Atherosclerosis, circRNA, Hsa_circ_0001879, HDAC9, miR-6873-5p, ceRNA

## Abstract

Atherosclerosis (AS) is a typical vascular disease. Emerging evidence has shown that circRNAs play key roles in the progression of AS, but the potential function and underlying mechanism of hsa_circ_0001879 remains unknown. We detected the expression level of hsa_circ_0001879 was determined by qRT-PCR, and the proliferation rate and migration ability of HUVECs were measured by CCK-8 assay and Transwell assay, respectively. Proliferative markers and epithelium mesenchymal transition (EMT) markers were measured through immunoblotting. A dual luciferase activity assay was performed to detect the interaction between circRNAs, miRNAs, and mRNAs. Hsa_circ_0001879 was upregulated in AS patients. Hsa_circ_0001879 inhibited the proliferation and migration ability of Human umbilical vein endothelial cells (HUVECs). Hsa_circ_0001879 directly bound to miR-6873-5p and acted as a sponge. miR-6873-5p-induced HDAC9 mRNA degradation was inhibited by hsa_circ_0001879. Hsa_circ_0001879 decreased the proliferation and migration of HUVECs by inhibiting miR-6873-5p-induced HDAC9 degradation.

## Background

Atherosclerosis (AS) is a major chronic disease characterized by the formation of atherosclerotic plaques, which pose a high risk to the cardiovascular system [1]. During the progression of AS, endothelial cells (ECs) and smooth muscle cells are the key agents regulating the progression of AS [2–4]. The pathogenesis of AS can be summarized as follows: an increase in oxidation of low density lipoprotein (ox-LDL), the dysfunction of ECs and an increase in the apoptosis of ECs caused by mitochondrial damage [5]. Discovering the potential mechanism of ox-LDL in the progression of AS will help us better understand its pathogenesis.

circRNAs are noncoding RNAs formed through the back-splicing of pre-mRNAs. circRNAs do not have a polyA tail or cap [6]. With the advancement of high-throughput sequencing, circRNAs were reported to be involved in the progression of AS [7]. Hsa_circ_0001445 reverses ox-LDL‑induced inhibition of HUVEC proliferation by targeting SRSF1 [8]. Inhibiting circ_UBR4 (hsa_circ_0010283) suppressed the proliferation, migration, and cell cycle progression of human vascular smooth muscle cells and promotes the progression of AS [9]. Circ_0004104 protects vascular endothelial cells from low-density lipoprotein-induced dysfunction by targeting the miR-328-3p/TRIM14 axis [10]. Circ_0093887 upregulates CCND2 and SUCNR1 expression to inhibit ox-LDL-induced endothelial cell dysfunction by functioning as a miR-876-3p sponge to compete with endogenous RNA [11], circ-SATB2 was upregulated in proliferating vascular smooth muscle cells (VSMCs) and regulated the phenotypic differentiation, proliferation, migration and apoptosis of VSMCs by miR-939-STIM1 axes [12], hsa_circ_0004104 was reported to regulated the macrophage function and was suggested as a potential therapy target for AS [13]. CicRNA circANRIL from the INK4/ARF locus was reported to regulate the expression of INK4/ARF and be associated with atherosclerotic vascular disease (ASVD) risk as well [14]. hsa_circ_0001879 was reported to be the biomarker of coronary artery disease [15], however, the potential function and underlying mechanism was still unknown.

In this study, we hypothesized that hsa_circ_0001879 was engaged in the progression of AS and we tried to uncover the underlying mechanism, thus, we evaluated the expression of hsa_circ_0001879 in AS patients and found that hsa_circ_0001879 was upregulated. After establishing stable cell lines, the proliferation rate and migration ability of the cells were detected by CCK-8 assay and Transwell assay, respectively. Hsa_circ_0001879 inhibited the proliferation and migration of HUVECs. A dual luciferase activity assay showed that hsa_circ_0001879 inhibited miR-6873-5p-induced HDAC9 degradation by acting as a competing endogenous RNA.

## Results

### Hsa_circ_0001879 was upregulated in AS patients and ox-LDL-treated HUVECs

We hypothesized that hsa_circ_0001879 was engaged in the progression of AS and we tried to uncover the underlying mechanism, thus, we evaluated the expression of hsa_circ_0001879 in AS patients and found that hsa_circ_0001879 was upregulated. After establishing stable cell lines, the proliferation rate and migration ability of the cells were detected by CCK-8 assay and Transwell assay, respectively. Hsa_circ_0001879 inhibited the proliferation and migration of HUVECs. A dual luciferase activity assay showed that hsa_circ_0001879 inhibited miR-6873-5p-induced HDAC9 degradation by acting as a competing endogenous RNA.

**Figure 1.**
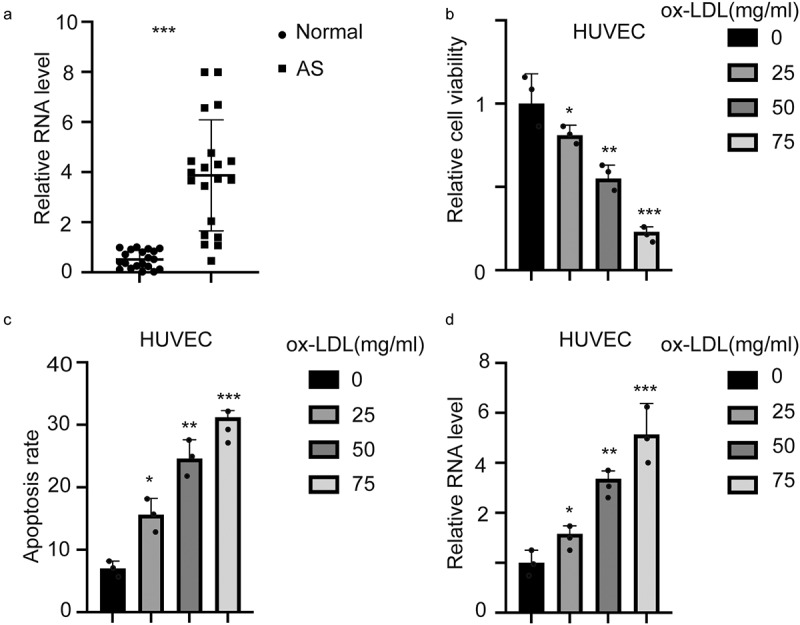
Hsa_circ_0001879 in upregulated in AS patients and ox-LDL-treated HUVECs. (a) 20 AS samples and 20 normal tissues were enrolled. The expression of hsa_circ_0001879 was measured and normalized. ***,p < 0.001. (b) HUVECs were treated with ox-LDL with different concentration (mg/ml). The relative cell viability and apoptosis rate (c) was measured, *, p < 0.05, **, p < 0.01, ***, p < 0.001.D, HUVECs were treated with ox-LDL and the relative level of hsa_circ_0001879 was detected and normalized, *, p < 0.05, **, p < 0.01, ***, p < 0.001

To detect the expression level of hsa_circ_0001879 in AS, we collected 20 samples from 20 AS patients and 20 normal tissues from volunteers. qRT-PCR was performed to measure the expression of hsa_circ_0001879. The results are shown in Figure 1a. Hsa_circ_0001879 expression was upregulated in AS patients. We next treated HUVECs with ox-LDL at different concentrations (mg/ml), and changes in cell viability (Figure 1b,*,p < 0.05,**,p < 0.01,***,p < 0.001) and the apoptosis rate were measured(Figure 1c,*,p < 0.05,**,p < 0.01,***,p < 0.001). Cell viability decreased with the application of ox-LDL, and the apoptosis rate increased. The expression level of hsa_circ_0001879 was increased in ox-LDL treated HUVECs in dosage dependent manner (Figure 1d,*,p < 0.05,**,p < 0.01,***,p < 0.001).

### Hsa_circ_0001879 inhibited the proliferation and migration of ox-LDL-treated HUVECs

**Figure 2.**
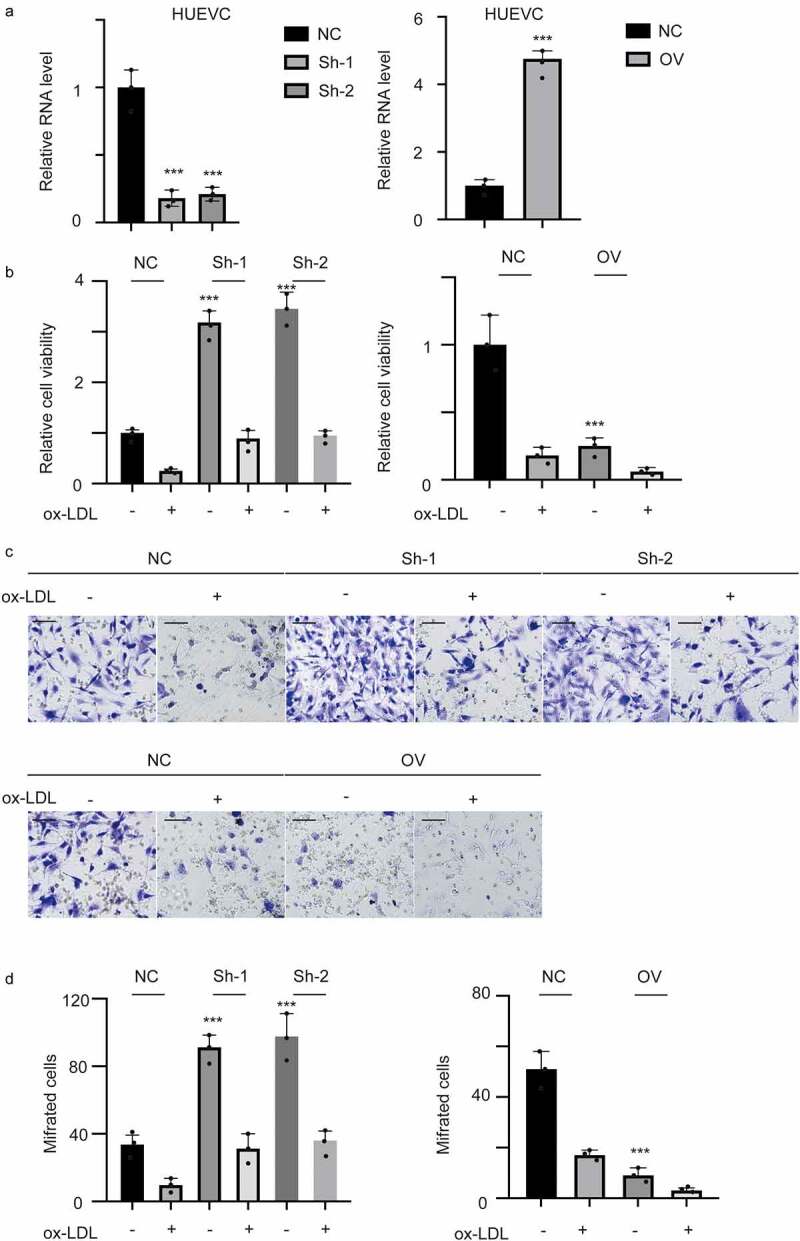
Hsa_circ_0001879 inhibits proliferation and migration of HUVECs. (a) we established hsa_circ_0001879 knocking down cell line using junction specific shRNA and overexpression cell line. hsa_circ_0001879 was detected and normalized to control, ***, p < 0.001. (b) after establishing stable cell line, ox-LDL was added (100 mg/ml) and relative cell viability was detected. (c) representative image of transwell assay of different cell lines.D, the statistical analysis of cells with indicated modifications in HUEVCs in (c), ***, p < 0.001

To determine the biology and underlying mechanism of hsa_circ_001879, we established stable overexpression and knockdown cell lines. The results are shown in Figure 2a (***, p < 0.001). We next detected the viability and migration ability of ox-LDL-treated HUVECs (100 mg/ml). The results showed that ox-LDL inhibited the proliferation (Figure 2b, p < 0.001) and migration of HUVECs (Figures 2c,Figures 2d, *, p < 0.05, **, p < 0.01, ***, p < 0.001). This inhibition was completely relieved by knocking down hsa_circ_0001879. Overexpression of hsa_circ_0001879 had a cooperative effect with ox-LDL.

### Hsa_circ_0001879 inhibited the expression of proliferation markers and the EMT

**Figure 3.**
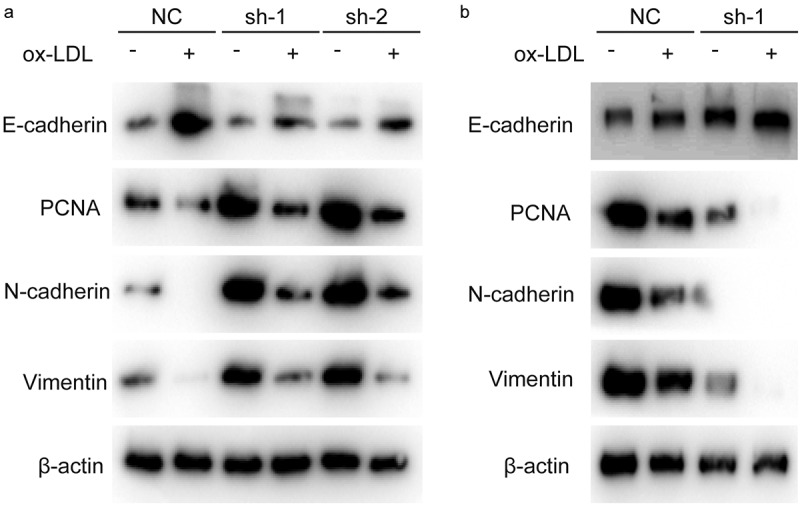
Hsa_circ_0001879 promotes the expression of PCNA and EMT progress. Cells with indicated modification was collected and subjected to immunoblotting, PCNA and EMT markers were measured

After showing that hsa_circ_0001879 inhibited the proliferation and migration of HUVECs, we measured the expression of PCNA and EMT markers, which are key markers reflecting the proliferation status and migration ability of cells. The results showed that PCNA expression was decreased in ox-LDL-treated cells and was restored in sh-hsa_circ_0001879 cells and decreased in hsa_circ_0001879-overexpressing cells (Figure 3).

### Hsa_circ_0001879 directly targeted miR-6873-5p as a competing endogenous RNA

**Figure 4.**
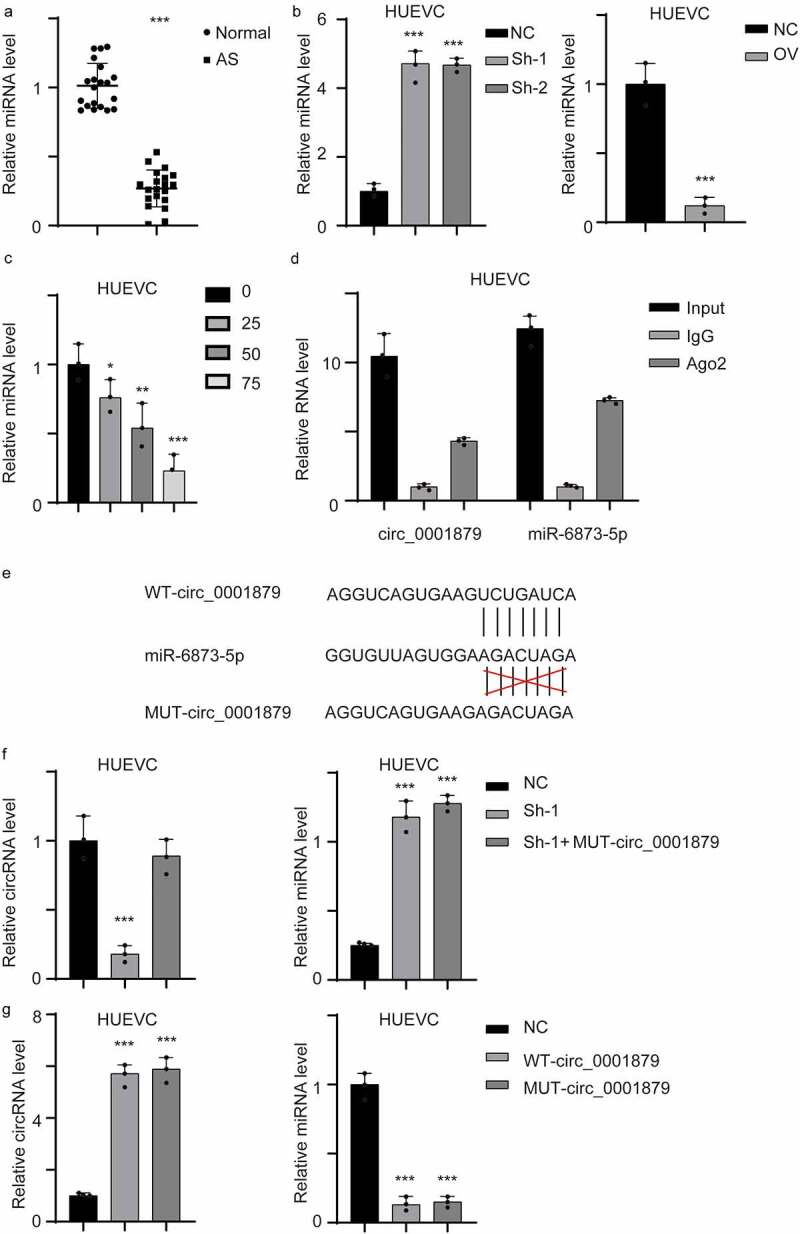
Hsa_circ_0001879 interacted with miR-6873-5p and act as compete endogenous RNA. (a) the miRNA-6873-5p level of the whole in-house cohort was detected and normalized, ***, p < 0.001. (b) the miRNA-6873-5p in different cell lines was detected and normalized, ***, p < 0.001. (c) HUVECs were treated with ox-LDL with different concentration (mg/ml), the relative miRNA level was detected, *, p < 0.05, **, p < 0.01, ***, p < 0.001. (d) RIP assay using AGO2 antibody and control IgG was applied. Relative hsa_circ_0001879 and miR-6873-5p level was detected. (e) the interaction residue between miR-6873-5p and hsa_circ_0001879 was shown and mutant allele was established accordingly. (f) We re-express MUT- circ_0001879 described in E in HUEVCs knocking down cell lines. Relative circ_0001879 level (left) and miRNA level (right) were detected, ***, p < 0.001. (g) We overexpress MUT- circ_0001879 and WT- circ_0001879 in HUEVCs. Relative circ_0001879 level (left) and miRNA level (right) were detected, ***, p < 0.001

circRNAs exerts their functions by acting as competing endogenous RNAs (ceRNAs). We searched the circRNA database and identified miR-6873-5p as a potential target miRNA. We first measured the expression of miR-6873-5p in AS patient samples. The results showed that miR-6873-5p was downregulated in the samples obtained from AS patients (Figure 4a, ***, p < 0.001). We next measured miR-6873-5p in the stable cell line and ox-LDL-treated HUVECs (Figures 4b,4c, *, p < 0.05, **, p < 0.01, ***, p < 0.001). miR-6873-5p expression was upregulated in hsa_circ_0001879-overexpressing cells but was decreased in hsa_circ_0001879-knockdown cell lines. The interaction between circRNAs and miRNAs was mediated by Ago2. We next performed a RIP assay and detected hsa_circ_0001879 and miR-6873-5p. The results showed that hsa_circ_0001879 and miR-6873-5p were present in the protein-RNA complex (Figure 4d). We next established a mutant (mut) hsa_circ_0001879 allele. We re-transfected the mut allele into the hsa_circ_0001879-knockdown cell lines and overexpressed the mut allele in HUVECs (figure 4f, ***, p < 0.001). The results showed that miR-6873-5p was not restored in the mut allele-re-expressing cell lines and that cells transfected with mut hsa_circ_0001879 had no influence on miR-6873-5p, (Figure 4g, ***,p < 0.001).

### Hsa_circ_0001879 inhibited miR-6873-5p-induced HDAC9 degradation

**Figure 5.**
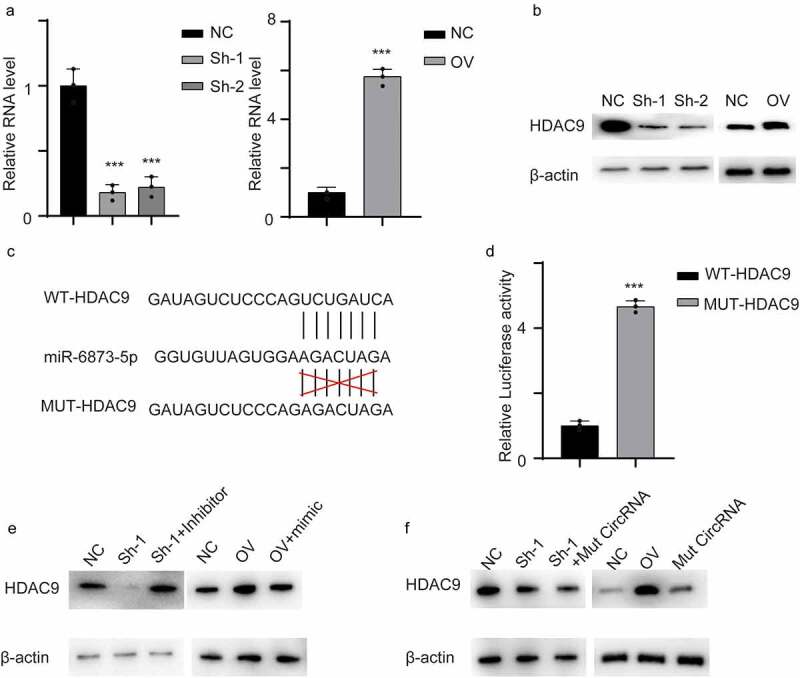
Circ_0001879 inhibited miR-6873-5p induced HDAC9 degradation. (a) the relative mRNA of HDAC9 in cells with indicated modifications. (b) immunoblot was used to detect HDAC9 in different cell line. (c) the interacting residue in HDAC9 and miR-6873-5p described in TargetScan. (d) we transfected dual luciferase reporting plasmid into HEK293T cell lines with WT-HDAC9 and MUT-HDAC9 accordingly. Relative luciferase activity was detected. (e) We applied miR-6873-5p inhibitor in circ_0001879 knocking down cell lines and miR-6873-5p mimic in circ_0001879 overexpression cells, immunoblot was used to detect HDAC9. (f) We re-express MUT- circ_0001879 described in E in HUEVCs knocking down cell lines and overexpress MUT- circ_0001879 and WT- circ_0001879 in HUEVCs. Immunoblot was used to detect HDAC9

By searching TargetScan, we identified HDAC9 as a potential target gene of miR-6873-5p. We first measured HDAC9 mRNA levels in cell lines. HDAC9 was decreased in hsa_circ_0001879-knockdown cells at both the mRNA and protein levels (Figures 5a,Figures 5b, ***, p < 0.001). The potential binding site of HDAC9 and miR-6873-5p is shown in Figure 5c. We performed a dual luciferase activity reporting assay. The results showed that the luciferase activity increased in the MUT-HDAC9 cells compared with the control WT-HDAC9 cells, and this result further confirmed the interaction (Figure 5d, ***, p < 0.001). We next transfected a miR-6873-5p inhibitor into the hsa_circ_0001879-knockdown cell line and a miR-6873-5p mimic into hsa_circ_0001879-overexpressing cells. The results showed that the expression of HDAC9 was completely restored in both cell lines(Figure 5d). We next measured HDAC9 expression in cells cotransfected with mut hsa_circ_0001879. HDAC9 remained unchanged in the cells transfected with mut hs_circ_0001879. The results indicated that hsa_circ_0001879 inhibited miR-6873-5p-induced HDAC9 degradation(Figure 5e).

## Methods

Patient information and clinical sample collection

AS samples and normal tissues were randomly collected from patients in the department of Cardiology, Guangdong Provincial Hospital of Traditional Chinese Medicine; The Second Affiliated Hospital of Guangzhou University of Chinese Medicine. The including criteria was detailed as below: samples from patients with peripheral vascular AS were included and the samples were confirmed by pathologists. The normal tissues were normal peripheral vascular tissues from donors. This study was approved by the Ethics Committee of The Department of Cardiology, Guangdong Provincial Hospital of Traditional Chinese Medicine; The Second Affiliated Hospital of Guangzhou University of Chinese Medicine. Clinical samples were collected from patients after written and informed consent was obtained.

### Stable cell line construction

The hsa_circ_0001879 plasmid was generated by chemical synthesis of the complete sequence of hsa_circ_0001879, and additional circularization promoter ALU sequences were added upstream and downstream, the sequence was detailed as below: 5ʹTCTGACAACTGAACTGCTCTCGCCTTGAACCTGTTTTGGCACTAA AATAAAATCTGTTCAATTAACGAATTCTGAAATATGCTATCTTACAG -GTGAATATATTTTTTCTTGAGGATCCACTAATTTGGGATGATAACGCCAAAACAGGTTCAAGGCGAGAGCAGTTCAGTTGTCAGAA3ʹ, the sequence of hsa_circ_0001879 was cloned between AG and GT in the blank. The hsa_circ_0001879 shRNA-1 sequence was GGCTGTTCGGGAAAGTGTCAA, and the shRNA-2 sequence was CGGCTGTTCGGGAAAGTGTCA. The plasmids were transfected with Lipofectamine 3000 (Invitrogen, Carlsbad, CA, USA) according to the manufacturer’s instructions. After transfecting, cells were treated with puromycin for 3 days and stable cell lines were established.

### qRT-PCR analysis

Tissues and cells were harvested and lysed with TRIzol (Sigma 93,289) following the manufacturer’s protocol. After purification and reverse transcription, cDNA was harvested. Specific primers were used for gene amplification. qPCR was performed using the SYBR Green detection RT‑PCR system (Takara Bio, Inc.) with RT mix (cat. no., RR036B; Takara Bio, Inc.) and SYBR Green (cat.no., 740,703; Takara Bio, Inc.). The thermocycling conditions were as follows: initial denaturation at 95°C for 5 s; 40 cycles at 95°C for 5 s of denaturation, 95°C for 35 s and 60°C for 30 s for annealing and elongation and 60°C for 30 s for final extension; and GAPDH was used for normalization. RNA was examined by detecting the absorption of OD260/OD230, RNA with ratio: > 2 was retained.

The following key primers were used:

hsa_circ_0001879 (F) CCCTCCAAGTCCAGTAAGAAGT

hsa_circ_0001879 (R) AGATCCTAAGAGGTGCGAGTTTA

miR-6873-5p (F): CTTCTCTGTAAGGCAAAGT

miR-6873-5p (R): CTCTACAGCTATTCCGAAC

HDAC9 (F): AGTAGAGAGGCAT

HDAC9 (R): GGAGTGTCCTTTCGG

U6 (F): CTCGCTTCGCAGCACA

U6 (R): AACGCTTCACGAATTTGCG

GAPDH (F) GGAGCGAGATCCCTCCAAAAT

GAPDH (R) GGCTGTTGTCATACTTCTCATGG

### Cell culture

Human umbilical vein endothelial cells (HUVECs) were acquired from the American Type Culture Collection. Cells were cultured with complete F12K (Sigma D0697) containing 10% fetal bovine serum (Invitrogen F8687). The cells were treated with different concentrations of ox-LDL for 48 h.

### Western blotting

The cells were harvested and washed three times with ice-cold PBS and lysed with RIPA buffer (Sigma 20–188). The protein was quantified using a BCA kit (Beyotime Institute of Biotechnology) according to the manufacturer’s protocol. After quantification, equal amounts of protein were separated by SDS-PAGE. Then, the proteins were transferred onto a polyvinylidene fluoride membrane and blocked with 5% milk for 1 h at room temperature. The membrane was incubated overnight with primary antibodies at 4°C, washed with TBST and then incubated with secondary antibodies (1:10,000; cat. no. 5724, KPL, Inc.) for 1 h at room temperature. Target proteins were detected using the ECL (EMD Millipore, MA, USA) method. The following antibodies were used:

anti-PCNA (ab18197, Abcam, 1:1000), anti-β-actin (CST, #3700, 1:1000), anti-N-cadherin (CST, #13,116, 1:1000), anti-E-cadherin (CST, #14,472, 1:1000), and anti-vimentin (CST, #5741, 1:1000).

### Dual-luciferase reporter assay

The fragment of hsa_circ_0001879 or HDAC9 3′UTR containing the wild-type or mutant allele was inserted into the pmirGLO vector (LMAI Bio, Shanghai, China) to create a WT-hsa_circ_0001879, MUT-hsa_circ_0001879, WT-HDAC9, or MUT-HDAC9 reporter. The plasmids were transfected into HUVECs, and the relative luciferase activities were measured with a Dual-Lucy assay kit (Solarbio).

### RNA Immunoprecipitation (RIP) assay

RIP analysis was performed using an EZ-Magna RIP kit (Millipore, Billerica, MA, USA) following the manufacturer’s instructions. After lysing cells with RIP lysis buffer, cell lysates were incubated with magnetic beads coated with anti-Ago2 or anti-IgG (as the control). Additionally, qRT-PCR analysis was performed to measure the levels of hsa_circ_0001879 and miR-6873-5p.

### Statistical analysis

All data analyses were performed with SPSS 20.0 statistical software. The differences between two groups were compared by Student’s t-test. For multigroup analysis, one‑way ANOVA followed by Tukey’s post hoc test was performed when making comparisons within datasets. A p value <0.05 was considered to indicate a significant difference compared to the control.

## Discussion

Evidence has demonstrated that endothelial cell dysfunction contributes to the progression of atherosclerosis [16,17]. The ox-LDL-treated HUVEC model has been well-established [18].

In our study, we identified a novel circRNA induced by ox-LDL and inhibited the proliferation and migration of HUEVCs through directly binds with miR-6873-5p and finally inhibited the degradation of HDAC9, EMT of endothelium cells and smooth muscle cells contributes critically to the progression of AS and understanding the underlying mechanism helped us better understand the disease [19].

CircRNAs play key roles in the progression of AS, especially in the function of endothelium vein cells. Hsa_circ_0030042 regulates autophagy and protects atherosclerotic plaque stability by targeting eIF4A3 [20]. The circular RNA circ_0003204 inhibits the proliferation, migration and tube formation of endothelial cells in atherosclerosis via the miR-370-3p/TGFβR2/phosph-SMAD3 axis [21]. circGNAQ was reported to inhibit endothelial cell senescence and atherosclerosis progression [22].However, the potential mechanism of circRNA on endothelium cells was rarely studied.

In summary, circRNAs exert their functions in three main ways: as competing endogenous RNAs, RNA-binding protein partners and facilitators of peptide translation [23]. In the progression of AS, circRNAs mainly act as ceRNAs. Hence, we investigated the potential mechanism of hsa_circ_0001879 as a miRNA sponge. By investigating the circRNA database, we identified miR-6873-5p and HDAC9 as the potential target axis. Mutant hsa_circ_0001879 was established to confirm the interaction between hsa_circ_0001879 and miR-6873-5p. We next detected miR-6873-5p-induced HDAC9 degradation. When we re-expressed a miR-6873-5p inhibitor and a miR-6873-5p mimic, the change in HDAC9 level was completely restored, indicating that hsa_circ_0001879 targets miR-6873-5p and thereby inhibits HDAC9 degradation.

The absence of HDAC9 attenuated AS progression by reducing inflammation and reversing cholesterol transport [24]. HDAC9 has been reported to be an independent biomarker useful for predicting the risk of AS [25]. HDAC9 activates IKK and thus regulates atherosclerotic plaque vulnerability [26]. HDAC9 has also been reported to regulate E2F3 and Rb1 and the progression of AS [27]. Most importantly, HDAC9 has been shown to be critical for ox-LDL-induced endothelial dysfunction [28]. Understanding the potential mechanism of HDAC9 in AS and its upstream regulation is urgently needed.

In conclusion, hsa_circ_0001879 increased the expression of HDAC9 by sponging miR-6873-5p, thereby preventing the cell growth and migration of HUVECs. These findings suggest that hsa_circ_0001879 is a potential therapeutic target for AS.

## Conclusion

Our study indicated that hsa_circ_0001879 promotes the progression of AS through inhibit miR-6873-5p induced HDAC degradation by acting as a competing endogenous RNA.

## Supplementary Material

Supplemental Material

## Highlights

- Hsa_circ_0001879 promotes the progression of AS
- Hsa_circ_0001879 directly bound to miR-6873-5p and acted as a sponge
- Hsa_circ_0001879 inhibited miR-6873-5p-induced HDAC9 degradation

## Declaration

Ethics approval and consent to participate

Studies were performed conforming to the Declaration of Helsinki and received approval from the Ethics Committee of the department of Cardiology, Guangdong Provincial Hospital of Traditional Chinese Medicine; The Second Affiliated Hospital of Guangzhou University of Chinese Medicine. Clinical samples were collected from patients after written informed consent was obtained.

Consent for publication

Not applicable

## Availability

All data was available in this article.

## References

[1] Libby P, Buring JE, Badimon L, et al. Atherosclerosis. Nat Rev Dis Primers. 2019;5(1):56. 31420554 10.1038/s41572-019-0106-z

[2] Pan S. Molecular mechanisms responsible for the atheroprotective effects of laminar shear stress. Antioxid Redox Signal. 2009;11(7):1669–1682. 19309258 10.1089/ars.2009.2487 PMC2842586

[3] Madamanchi NR, Runge MS. Mitochondrial dysfunction in atherosclerosis. Circ Res. 2007;100(4):460–473. 17332437 10.1161/01.RES.0000258450.44413.96

[4] Mercer JR, Cheng -K-K, Figg N, et al. DNA damage links mitochondrial dysfunction to atherosclerosis and the metabolic syndrome. Circ Res. 2010;107(8):1021–1031. 20705925 10.1161/CIRCRESAHA.110.218966 PMC2982998

[5] Park HJ, Zhang Y, Georgescu SP, et al. Human umbilical vein endothelial cells and human dermal microvascular endothelial cells offer new insights into the relationship between lipid metabolism and angiogenesis. Stem Cell Rev. 2006;2(2):93–102. 17237547 10.1007/s12015-006-0015-x

[6] Circular RNA. Nat Biotechnol. 2021;39(1):23. 33432216 10.1038/s41587-020-00787-2

[7] Jiao S, Wu S, Huang S, et al. Advances in the identification of circular RNAs and research into circRNAs in human diseases. Front Genet. 2021;12:665233. 33815488 10.3389/fgene.2021.665233 PMC8017306

[8] Liang G, Chen S, Xin S, et al. Overexpression of hsa_circ_0001445 reverses oxLDLinduced inhibition of HUVEC proliferation via SRSF1. Mol Med Rep. 2021;24(1). DOI:10.3892/mmr.2021.12146. PMC8134882 33982782

[9] Zhang Y, Zhang C, Chen Z, et al. Blocking circ_UBR4 suppressed proliferation, migration, and cell cycle progression of human vascular smooth muscle cells in atherosclerosis. Open Life Sci. 2021;16(1):419–430. 33981849 10.1515/biol-2021-0044 PMC8085462

[10] Zhang C, Wang L, Shen Y. Circ_0004104 knockdown alleviates oxidized low-density lipoprotein-induced dysfunction in vascular endothelial cells through targeting miR-328-3p/TRIM14 axis in atherosclerosis. BMC Cardiovasc Disord. 2021;21(1):207. 33892646 10.1186/s12872-021-02012-7 PMC8066471

[11] Gao Y, Li G, Fan S, et al. Circ_0093887 upregulates CCND2 and SUCNR1 to inhibit the ox-LDL-induced endothelial dysfunction in atherosclerosis by functioning as a miR-876-3p sponge. Clin Exp Pharmacol Physiol. 2021;48(8):1137–1149. 33844344 10.1111/1440-1681.13504

[12] Mao YY, Wang J-Q, Guo -X-X, et al. Circ-SATB2 upregulates STIM1 expression and regulates vascular smooth muscle cell proliferation and differentiation through miR-939. Biochem Biophys Res Commun. 2018;505(1):119–125. 30241943 10.1016/j.bbrc.2018.09.069

[13] Wang L, Shen C, Wang Y, et al. Identification of circular RNA Hsa_circ_0001879 and Hsa_circ_0004104 as novel biomarkers for coronary artery disease. Atherosclerosis. 2019;286:88–96. 31103880 10.1016/j.atherosclerosis.2019.05.006

[14] Burd CE, Jeck WR, Liu Y, et al. Expression of linear and novel circular forms of an INK4/ARF-associated non-coding RNA correlates with atherosclerosis risk. PLoS Genet. 2010;6(12):e1001233. 21151960 10.1371/journal.pgen.1001233 PMC2996334

[15] Wu N, Li J, Chen X, et al. Identification of long non-coding RNA and circular RNA expression profiles in atrial fibrillation. Heart Lung Circ. 2020;29(7):e157–e167. e157-e167. 31843366 10.1016/j.hlc.2019.10.018

[16] Tousoulis D, Simopoulou C, Papageorgiou N, et al. Endothelial dysfunction in conduit arteries and in microcirculation. Novel therapeutic approaches. Pharmacol Ther. 2014;144(3):253–267. 24928320 10.1016/j.pharmthera.2014.06.003

[17] Davignon J, Ganz P. Role of endothelial dysfunction in atherosclerosis. Circulation. 2004;109(23 Suppl 1):III27–32. 15198963 10.1161/01.CIR.0000131515.03336.f8

[18] Lu J, Mitra S, Wang X, et al. Oxidative stress and lectin-like ox-LDL-receptor LOX-1 in atherogenesis and tumorigenesis. Antioxid Redox Signal. 2011;15(8):2301–2333. 21338316 10.1089/ars.2010.3792

[19] Sorokin V, Vickneson K, Kofidis T, et al. Role of vascular smooth muscle cell plasticity and interactions in vessel wall inflammation. Front Immunol. 2020;11:599415. 33324416 10.3389/fimmu.2020.599415 PMC7726011

[20] Yu F, Zhang Y, Wang Z, et al. Hsa_circ_0030042 regulates abnormal autophagy and protects atherosclerotic plaque stability by targeting eIF4A3. Theranostics. 2021;11(11):5404–5417. 33859754 10.7150/thno.48389 PMC8039966

[21] Zhang S, Song G, Yuan J, et al. Circular RNA circ_0003204 inhibits proliferation, migration and tube formation of endothelial cell in atherosclerosis via miR-370-3p/TGFbetaR2/phosph-SMAD3 axis. J Biomed Sci. 2020;27(1):11. 31900142 10.1186/s12929-019-0595-9 PMC6941276

[22] Wu WP, Zhou MY,Liu DL, et al. circGNAQ, a circular RNA enriched in vascular endothelium, inhibits endothelial cell senescence and atherosclerosis progression. Mol Ther Nucleic Acids. 2021;26:374–387. 34552819 10.1016/j.omtn.2021.07.020 PMC8426466

[23] He AT, Liu J, Li F, et al. Targeting circular RNAs as a therapeutic approach: current strategies and challenges. Signal Transduct Target Ther. 2021;6(1):185. 34016945 10.1038/s41392-021-00569-5 PMC8137869

[24] Schiano C, Benincasa G, Franzese M, et al. Epigenetic-sensitive pathways in personalized therapy of major cardiovascular diseases. Pharmacol Ther. 2020;210:107514. 32105674 10.1016/j.pharmthera.2020.107514

[25] Qingxu G, Yan Z, Jiannan X, et al. Association between the gene polymorphisms of HDAC9 and the risk of atherosclerosis and ischemic stroke. Pathol Oncol Res. 2016;22(1):103–107. 26347468 10.1007/s12253-015-9978-8

[26] Asare Y, Campbell-James T, Bokov Y, et al. Histone deacetylase 9 activates IKK to regulate atherosclerotic plaque vulnerability. Circ Res. 2020;127(6):811–823. 32546048 10.1161/CIRCRESAHA.120.316743

[27] Prestel M, Prell-Schicker C, Webb T, et al. The atherosclerosis risk variant rs2107595 mediates allele-specific transcriptional regulation of HDAC9 via E2F3 and Rb1. Stroke. 2019;50(10):2651–2660. 31500558 10.1161/STROKEAHA.119.026112

[28] Han X, Han X, Wang Z, et al. HDAC9 regulates ox-LDL-induced endothelial cell apoptosis by participating in inflammatory reactions. Front Biosci (Landmark Ed). 2016;21:907–917. 27100479 10.2741/4428

