# Supplementary figures and images for: Hsa_circ_0001879 promotes the progression of atherosclerosis by regulating the proliferation and migration of oxidation of low density lipoprotein (ox-LDL)-induced vascular endothelial cells via the miR-6873-5p-HDAC9 axis
Source: Bioengineered. 2021 Dec 7;12(2):10420–9. doi: 10.1080/21655979.2021.1997224 (PMC8809926; doi:10.1080/21655979.2021.1997224)

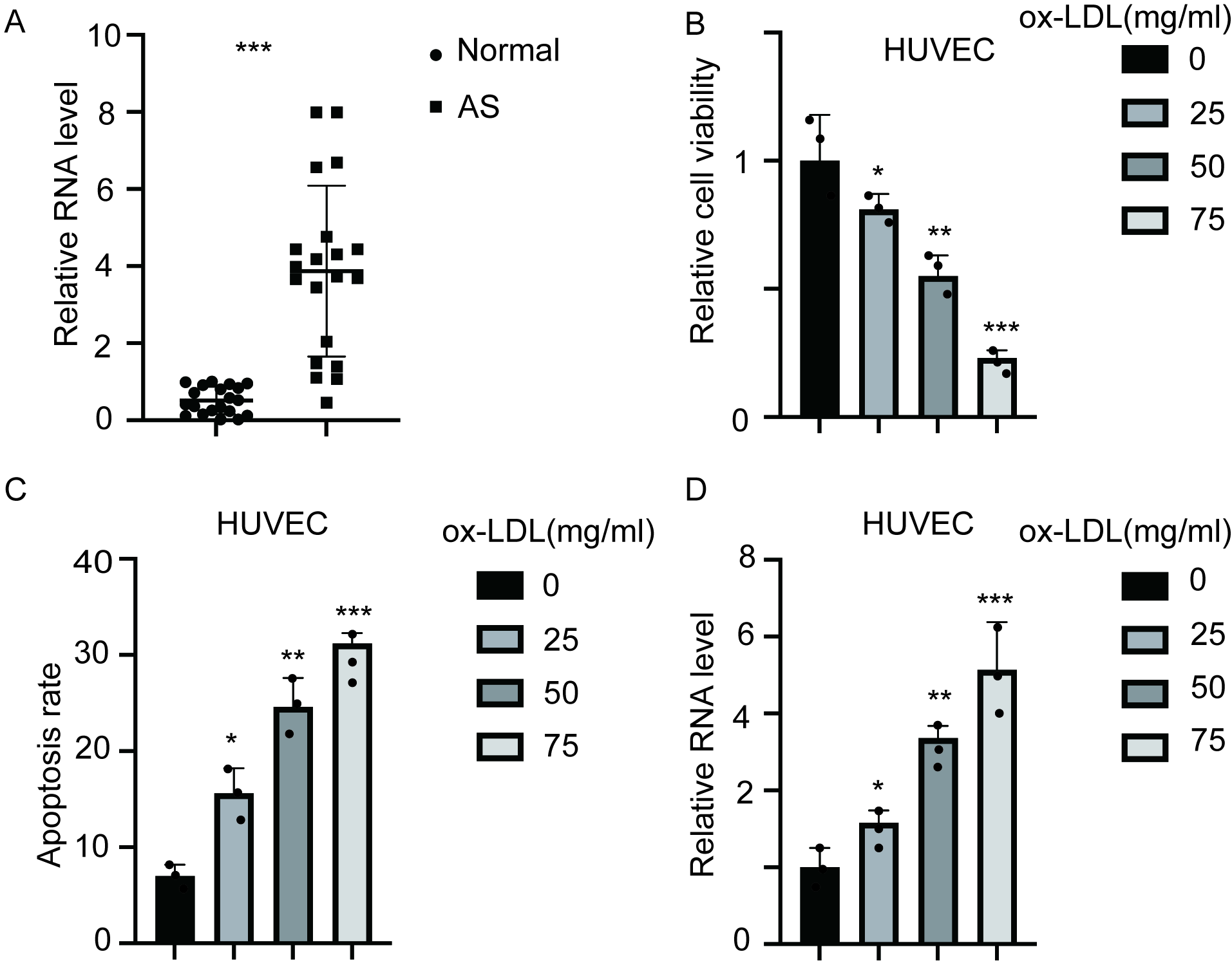

Supplement: Supplemental Material [file KBIE_A_1997224_SM6778.zip › supplementary/sF1.tif]

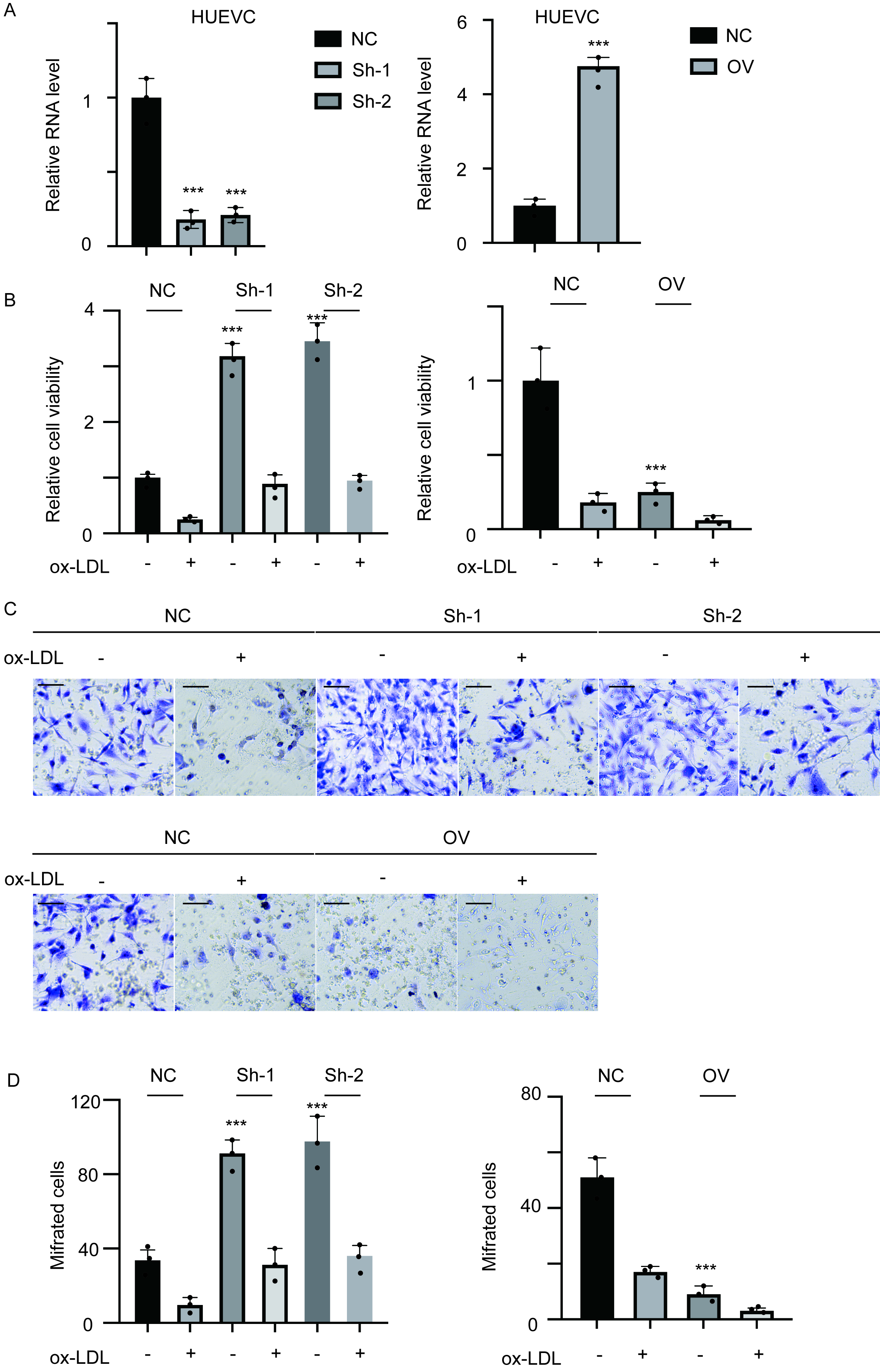

Supplement: Supplemental Material [file KBIE_A_1997224_SM6778.zip › supplementary/sF2.tif]

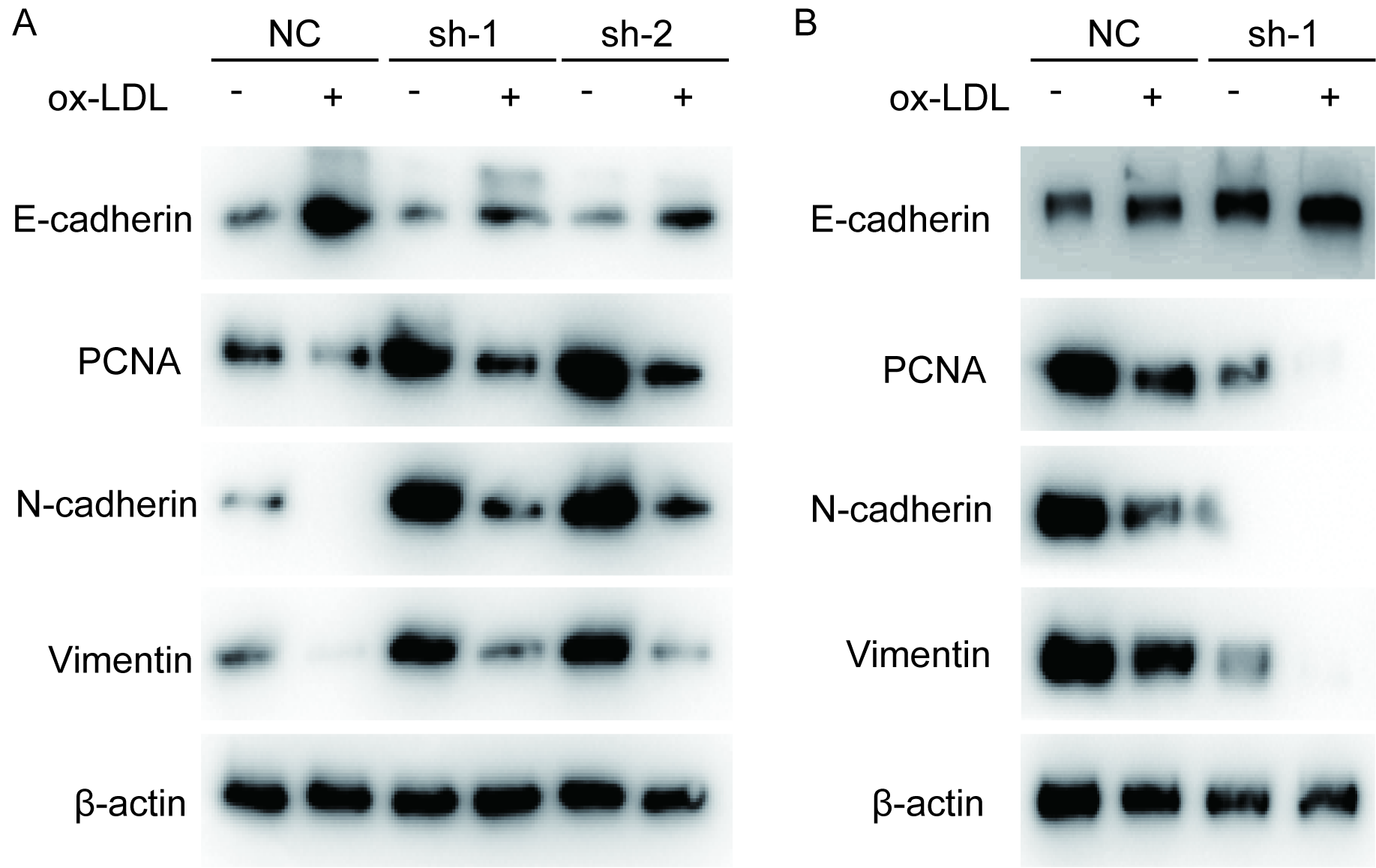

Supplement: Supplemental Material [file KBIE_A_1997224_SM6778.zip › supplementary/sF3.tif]
